# Evaluation of Education, Attitude, and Practice of the Turkish Anesthesiologists in Regional Block Techniques

**DOI:** 10.5812/aapm.7632

**Published:** 2013-03-26

**Authors:** Hakan Baydar, Leyla Seden Duru, Sevda Ozkardesler, Mert Akan, Reci Dalak Meseri, Gozde Karka

**Affiliations:** 1 Clinic of Anesthesiology, Carsamba State Hospital, Samsun, Turkey; 2 Department of Anesthesiology, School of Medicine, Dokuz Eylul University, Izmir, Turkey; 3 Department of Nutrition and Dietetics, College of Health Sciences, Ege University, Izmir, Turkey

**Keywords:** Anesthesia, Conduction, Education, Professional Practice

## Abstract

**Background:**

The demand for regional blocks from both patients and surgeons has significantly increased in anesthesia practice during the last 30 years. Although the studies show that the complications are rare, regional blocks still have serious difficulties which can be prevented by training programs.

**Objectives:**

The purpose of this study was to determine the factors affecting the educational methods, attitude and practice of the Turkish anesthesiologists in regional blocks during and following residency programs.

**Patients and Methods:**

Anesthesiologists were asked to answer a questionnaire. Educational proficiency was determined by at least 50 spinal, 50 epidural and 50 peripheral block applications during residency. Specialists were asked for the numbers of spinal, epidural and peripheral blocks (PBs) they applied in 2009. The mean and median values were calculated.

**Results:**

One hundred and eighty-eight anesthesiologists (84.3 %) agreed to participate in the study. While all participants had made their first attempts in neuraxial blocks (NBs) when they were residents, this ratio was detected as 96.8% for PBs. All participants learned neuraxial and PBs on patients in the operating theater. Education proficiency ratios for spinal, epidural and PBs were 98.1 %, 92.5 % and 62.3 %, respectively. Age, perception of adequate training, nerve block rotation, adequate application in education, following innovations were the factors which significantly affected the number of PBs in practice according to univariate analysis. The participants who consider their applications on NBs were adequate (P = 0.029) and the ones working in state or private hospitals (P = 0.017), applied NBs significantly above the median number.

**Conclusions:**

Anesthesiologists had adequate education and practice of NB applications but a significant proportion of participants (51.8%) lacked both in PBs applications. We believe that NBs are more easily learned than PBs during residency training program.

## 1. Background

The demand for regional blocks (RBs) from both patients and surgeons has significantly increased in anesthesia practice during the last 30 years (1, 2). Although the studies show a reduction in attendant complications, there are still some serious ramifications of these techniques which can be prevented by adequate training programs (3, 4). There are some studies on these training programs in USA and Canada which endower to educate the residents on the applications, indications, contraindications and complications of these techniques. Teaching methods like cadaver workshops, electronic models and ultrasound-guided regional anesthesia are recommended to improve the quality of the techniques (1, 5). According to the Residency Review Committee for Anesthesiology (RRCA) in the United States, residents should carry out at least 40 spinals, 50 epidurals and 40 unspecified peripheral blocks (PBs) as well as 25 nerve blocks in pain management (6). Similarly, the German Society for Anesthesia and Intensive Care (DGAI) demands 100 neuraxial blocks (NBs) and 50 PBs during residency (7). Turkish Anesthesiology and Intensive Care Society (TAICS) Adequacy Committee has taken the suggestions of European Anesthesiology Adequacy Committee (EAAC) as a model and designs residency training based on these suggestions. During five years of training the minimum recommended target numbers for RB techniques are 50 for spinal anesthesia (SA), 50 for epidural anesthesia (EA) and 50 for PBs. However, training time for regional anesthesia (RA), required educational tools for the process, details such as how to evaluate resident’s performance and success are not presented in detail in this report.

## 2. Objectives

The goal of this study was to determine the factors affecting the educational methods, attitude and practice of the Turkish anesthesiologists in RBs during and after residency.

## 3. Patients and Methods

This study was approved by the Medical Faculty Ethics Committee of Non-Invasive Clinical Research. All of the anesthesiologists (n = 228) who work in state, private and university hospitals of Izmir according to the data of the Turkish Health Ministry were invited to participate in the study in 2010. Our objective was to reach all anesthesiologists face-to-face without sampling. The exclusion criteria were: completion of the residency in a foreign country and being unreachable in his or her hospital. The anesthesiologists were asked to fill a questionnaire which consisted of 4 parts and 49 questions. In the first section, demographic and educational variables of the participants were evaluated, whereas in three other sections, the features of the practical applications (within the last year), including prior to the block, rendering stage of the block and after the block had been applied, were investigated. Following informed consent, the questionnaire was given to the anesthesiologist without getting his or her name in order to reach anonymous feedback and it was put in a closed box following completion. The dependent variable was the number of blocks they applied during the last year while the independent variables were: age, gender, the institution where the residency completed, their present institution, the rotations and courses they took about regional blocks, the degree to which they followed innovations, the use of assistant tools, the perception of adequacy in training and practice and adequacy in training. In this study, at least 50 spinal, 50 epidural and 50 PBs were needed for the baseline adequacy competence of RB training of an anesthesiology resident according to the EAAC model. If a specialist performed fewer than 50 procedures of each type during training period, we defined it as 'inadequate training' (or more than these numbers was accepted as 'adequate training'). We asked the specialists the numbers of spinal, epidural and PBs they applied within the last year, the mean and median values were calculated and the groups were separated according to the median values. Due to the large difference between the mean and median values and abnormal distribution of the samples, the median was chosen as the threshold value. Factors affecting the status of “applications are being done at median and above the median" were evaluated. Statistical analysis was performed by using SPSS version 15.0 (Statistical Package for the Social Sciences) software. Continuous data were presented as mean ± standard error (median, minimum-maximum) and the categorical data presented with percentages. Yates-corrected chi-square test was used for univariate analysis. P value less than 0.05 was considered statistically significant.

## 4. Results

**Figure 1:**
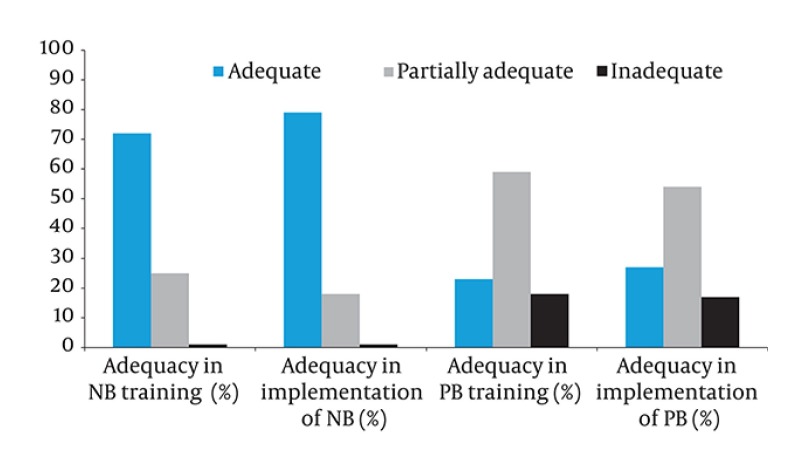
Participants’ Perceptions of Competencies in Training and Implementation of Neuraxial and Peripheral Blocks in Percentages

**Figure 2:**
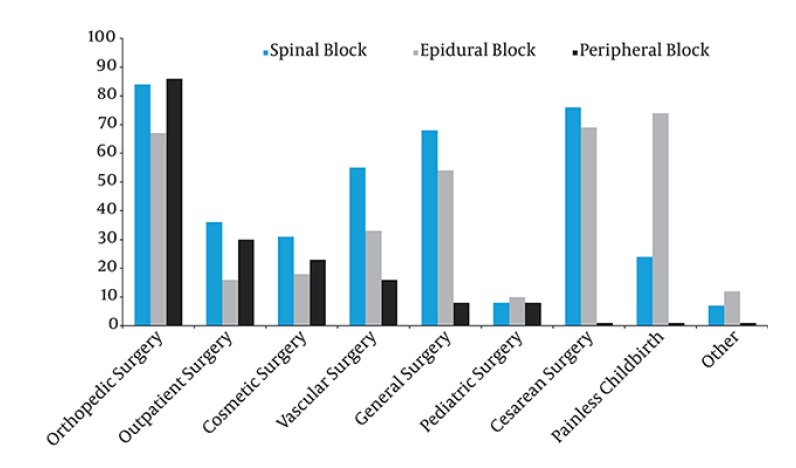
Frequencies of Spinal, Epidural and Peripheral Blocks Preferred in Different Surgeries

There were 228 anesthesiologists working in Izmir in 2010. Five anesthesiologists got their residency abroad; therefore the 223 of them were included in the study. Thirty-four (15.2%) anesthesiologists who could not be reached and the one (0.5%) who did not fill the questionnaire were excluded. One hundred and eighty-eight (84.3%) of 223 anesthesiologists agreed to participate in the study. The distribution of gender was similar (47.3% male, 52.7% female). The mean age of participants was 42.2 ± 6.7 (41.5, 30-65) years. One hundred and thirty six participants (72.3%) had completed residency training in university hospitals. Six anesthesiologists (3.2%) did not perform any PBs. While all participants (100%) had made their first attempts in NBs when they were residents, this ratio was detected as 96.8% for PBs. The universal way of learning NBs and PBs for all of the participants was on the patient in the operating theater. As the second most common form of learning was on the web sites (42 participants, 22.3%), the third and fourth forms, respectively, were the dissection of cadavers (18 participants, 9.6%) and applied studies in workshops (17 participants, 9.0%). The distribution of anesthesiologists' first attempts on NBs and PBs according to the years of their residency is presented in Table 1 . One hundred and fourteen participants (60.6%) reported that they learned RBs more easily in their residency program as they did not have direct responsibility. One hundred and forty three (76.1%) participants stated that they applied RBs more easily following they became a specialist as their knowledge and experience was greatly enhanced over their residency period. Sixteen of the participants (8.5%) got a rotation for RB in the institutions in which they were educated and those rotations lasted between two and eight months. Forty participants (21.3%) attended courses for RBs held within TAICS, while 36 of them (90.0%) preferred courses for PBs. Forty-four anesthesiologists (23.4%) attended some other courses. We determined that 151 participants (80.3%) followed the innovations related to regional blocks. For following innovations, 68.1% of them attended scientific events, where 66.5% of them followed printed material. More than half (54.3%) stated that they followed web sites and only a few of them went to another domestic center (9.6%) or abroad (1.1%). Six participants (3.2%) used an assistive device which 5 of them used a scopy, 1 of them used USG while performing NBs. Participants mostly preferred a nerve stimulator technique in PBs (171 participants answered the question, 106 (62%) of them preferred a nerve stimulator. Participants' perceptions of competencies in training and implementation of NBs and PBs are presented as percentages in Figure 1 ('partially adequate' option was considered as 'inadequate' in statistical analysis). One hundred and eighty-one of 187 respondents (96.8%) preferred regional anesthesia as the first choice in a patient for whom both regional and general anesthesia could have been applied. The reasons for not applying RBs revealed in Table 2, the surgeries in which they prefer spinal, epidural and PBs, respectively, are presented in Figure 2. Blocks were preferred 100% in elective surgeries, 60% in emergencies. Block application rate was very low in children that were awake (7.9%). The numbers of spinal, epidural and PBs that the participants applied during their training were 574.5 ± 58.8 (500, 0- 6500), 343.2 ± 35.3 (200, 0-3500), 171.5 ± 58.7 (50, 0-8000), respectively.

**Table 1:** The Distribution of Anesthesiologists’ First Attempts on Neuraxial and Peripheral Blocks According to the Years of Residency [Table-fn fn973]

Years of Residency	NB, No. (%)	PB, No. (%)
**1**	165 (87.8)	111 (63.1)
**2**	16 (8.5)	31 (17.6)
**3**	5 (2.7)	30 (17.00)
**4**	2 (1.1)	4 (2.3)
**Total**	188 (100.0)	176 (100.0)

Abbreviations: NB, neuraxial block; PB, peripheral block

**Table 2:** Reasons for not Applying Regional Blocks in Daily Practice

Reasons	Competency	
	Sufficient (n = 66), No. (%)	Insufficient (n = 71), No. (%)
**Not preferred by the patient**	51 (77.3)	54 (76.1)
**Not preferred by the surgeon**	12 (18.2)	29 (40.8)
**Worries about extension of time of surgery**	17 (25.8)	16 (22.5)
**Lack of material**	11 (16.7)	16 (22.5)
**Fear of failure**	4 (6.1)	22 (31.0)
**Medicolegal concerns**	6 (9.1)	20 (28.2)
**The lack of knowledge**	5 (7.6)	4 (5.4)
**The risk of passing to general anesthesia**	8 (12.1)	11 (15.5)
**Other**	20 (30.3)	4 (5.6)

### 4.1. Peripheral Block Practice

One hundred and thirty-seven of 188 participants (72.8%) responded to the question asking the number of PBs they applied during the past year. While 66 of 137 participants (48.2%) reported as median as and higher than the median number of blocks, 71 of them (51.8%) reported numbers lower than the median. Age above 40 years (P = 0.003), training adequacy (P = 0.023), perception of adequate education (P < 0.001) and application (P = 0.012), following innovations (P < 0.001) and PB rotation in another national center (P < 0.001) were the main factors which affected the participants’ peripheral block prectice significantly above the median in 2009. Participants who followed the innovations in printed materials (books, magazines etc.) (P = 0.03), with scientific events (congresses, symposiums, meetings etc.) (P < 0.001) and by internet sites (P = 0.02) applied PBs significantly above the median.

### 4.2. Neuraxial Block Practice

One hundred and sixty-six of 188 participants (88.3%) responded to the question asking the number of NBs they applied during last year. While 91 of 166 participants (54.8%) reported as median as and higher than median number of blocks, 75 of them (45.2%) reported numbers lower than the median. The participants who perceived their NB applications as adequate (P = 0.029) and the ones working in state or private hospitals (P = 0.017) applied NBs above the median significantly in 2009.

## 5. Discussion

The participation rate in the questionnaire was 84.3% in our study. Kopacz and Hadzic used questionnaires and reported participation rates, respectively, as 70.4% and 56.5% (4, 8). Chelly distributed the questionnaires through e-mail and fax and reported the responses rate as 52% (9). Unlike these investigators, we experienced a higher response rate which may have been due to the face-to-face interviews we encountered. The primary purpose of this survey was to evaluate the education of residents and their skills at the end of their training. For this reason, we wanted to determine whether the participants’ training had been adequate. We undertook the task to decide the adequacy of training according to the EAAC criteria. Educational adequacy rates in spinal, epidural and PB were, respectively, 98%, 92.5% and 62.3% in our study group. Based on these results, NB applications were adequate whereas PB applications were inadequate in many of the participants in the present study. These results show that NBs have been better assimilated than PBs during residency training. Various methods like written or oral tests have been developed to assess the important aspects of education such as cognitive information, decision-making, skills and adaptation. However, how and when they reached the level of competence in education of residents is still unclear (10). Our findings show that a great percentage of the participants applied their first NBs and PBs in the first 2 years of their residency and they had learned these skills on the patient in the operating theater. Kopacz et al. reported that RA is preferred in 30.2% of cases in current resident training in US (11). However, this rate showed a significant increase in 1980 (21.3%) but since 1990 (29.8%), no remarkable increase has been observed. We cannot give the distribution of the application rates of RA in our country according to the results of our study, but the participants primarily preferred to apply RBs at a rate of 96.8% in patients whom general and RA could be used alternatively which indicates the increase of demand for RA. Smith et al. evaluated the relation between self-confidence and the years of residency and they reported that the residents only felt confidence in applying lumbar epidural and spinal anesthesia at the end of their training period, but they found out a lack of confidence in other RB techniques (12). While Smith et al. had questioned the self-confidence of residents; we questioned the adequacy of their education and the implementation in their daily practice (12). Unlike other studies, we established that the participants, who thought their education and practice were adequate, applied PBs more than others. However, we identified that there was no effect of education adequacy on practice of NBs, but there was a significant impact of implementation adequacy in increasing the number of NB. In a survey study of Chelly et al., 65% of the programs had a specific PBs rotation period of 1 month which only one program had less than 1 month rotation (9). Very well-defined training schedule, a specialized team consisting of regional anesthesiologists and a well-structured rotation are the absolute pre-configured policies for adequate resident's education (6). Hadzic et al. described how a rotation of regional anesthesia should be and separated the techniques into three as basic, intermediate and advanced (6). According to this study, they taught basic techniques in the first two years of residency and on the last year of education they included basic and some intermediate level techniques, pharmacology of local anesthetics and principles of nerve stimulation, and also some advanced techniques in 2 month’s period. Our results demonstrated that the low rate of rotation is associated with a lack of well-defined rotation programs in the education system of our country. Despite this low rate of rotation, those who had a rotation showed a significant increase in the number of PBs in their daily practice indicating the importance of rotation in education. There are many factors that affect the personal and institutional training conditions such as institutional preferences, situation of learning and training, and the number of cases within the time period of training (13). In addition to the US, many European countries have reported a significant improvement in the quality of education in recent years; however, there are still shortcomings in education (7, 10). Buist stated that 33% of anesthesiologists still continue to learn RA in their 20-30 years of practices which emphasizes the need for permanency in education and follow-up of innovations (13, 14). Hargett et al. recommended a higher education program (fellowship) for RA for a period of at least one year following traditional education of anesthesia (5). Participants who followed the developments related to RA, applied more than median number of PBs in our study, which confirms the results of the other researchers. As stated by Chelly et al., we also concluded that the preferred method in PBs was the use of nerve stimulator (9). We also found that assistive device use had no effect on the number of blocks applied. Hadzic et al. reported that the anesthesiologists who work in educational institutions applied PBs significantly more than the ones work in other institutions (8). Unlike this study, we determined that there was no effect of institutions; they were educated or they worked on the implementations of PBs, although we identified that the anesthesiologists working in state or private hospitals had applied NBs more than the others. This difference is due to the fact that the number of PBs applications was smaller than the NBs applications. Perhaps, if the sample size had been increased for the variables of the institutions they were educated or worked, the results could have been different. The result of this study was different from Hadzic et al.'s study as the increasing age had a significant effect on the number of PBs applied (8). This result suggests the contribution of experience and developed hand skills on PBs implementation. Clergue et al. showed that RA was performed in 23% of all surgical cases and orthopedic procedures represented 44% of all cases (2). In the literature, it was reported that RA was used also more frequently in vascular, plastic and outpatient surgery (2, 8, 9). Similarly, we found that spinal anesthesia and PBs were frequently preferred for orthopedic surgery. The choice of regional anesthesia for emergency surgeries is reported by to be 19% (2), but we identified a rate of 61% which is above our expectations. This result revealed that the tendency to use RA is high in our country and as a reflection of this; it is more preferable in urgent cases. Residents have little knowledge of RA techniques when they commence their education and this causes low success rates and loss of self-confidence at the beginning. However, the number and the quality of the implementations increase after they gain the basic knowledge and develop their manual skills (15). In accordance with our results, we concluded that the anesthesiologists applied these techniques more with the increasing knowledge, but if they had insufficient knowledge and skills, they failed to learn some techniques easily and did not use them in their practice. The factors affecting the applications of RBs are the demands and the expectations of the patients, surgeons' behaviors and timing (4) as well as patient's satisfaction and time are the effective factors to decide the type of block to apply (12). Hanna et al. investigated the frequency and causes of not applying RBs in a study and established the first common cause as anesthesiology-dependent, second as surgeon-dependent and third as medical contraindications (16). According to our results, the patient's preference took the highest priority, whereas the surgeon and the time-related reasons took the second and third place. We consider the greatest limiting factor in the present study which was sample size. If we had included all the anesthesiologists in all cities, different results could have been obtained as they relate to anesthesiologists’ education and daily practice. In addition, it might be difficult for some anesthesiologists to remember their retroactive applications of anesthesia as the participants had different periods of work time. According to our results, anesthesiologists had adequate education and practice of NB applications but a significant proportion of participants (51.8%) lacked in PB applications. In addition, the adequacy in learning and applying blocks and following the innovations had a positive effect on PBs applications following graduating and setting up a practice. We believe that NBs are more easily learned than PBs during residency training program. Therefore, reviewing training programs of each educational institution, determining the number of applications for each type of PBs as well as NBs, specialized educational teams for RA, a defined rotation for RA and continuing vocational training following residency are required. Moreover, It is unknown why NBs were applied more in state and private hospitals as compared to teaching hospitals and the reason for such should be investigated.
